# Extrapulmonary sequestration with a left internal thoracic arterial feeding vessel in an infant treated with video-assisted Thoracoscopic resection: a case report

**DOI:** 10.1186/s13019-018-0775-9

**Published:** 2018-07-20

**Authors:** Laura DiChiacchio, Clint D. Cappiello, Jose Greenspon

**Affiliations:** 1 Department of Surgery, Univeristy of Maryland Medical Center, Baltimore, 21201 USA; 2 Department of Surgery, SSM Health Cardinal Glennon Children’s Hospital, St. Louis, 63104 USA

**Keywords:** Extrapulmonary sequestration, Video-assisted thoracoscopic surgery, Pediatric, Internal thoracic artery

## Abstract

**Background:**

Congenital lung malformations exist along a spectrum of pathogenesis and disease severity. Extrapulmonary sequestration (EPS), in which nonfunctional lung tissue develops without connection to the tracheobronchial tree, is one rare manifestation of this disease. Atypical vascular anatomy with a systemic feeding vessel characterizes these lesions.

**Case presentation:**

A 3 day old, 37 week gestation infant underwent chest X-ray for confirmation of umbilical catheter placement and was found to have an elevated left hemidiaphragm consistent with eventration versus congenital diaphragmatic hernia. He remained asymptomatic and was evaluated as an outpatient at the age of 9 months, where CT angiogram demonstrated extrapulmonary versus intrapulmonary sequestration with a systemic feeding vessel from the left internal mammary artery.

**Conclusions:**

It is exceedingly rare for the feeding artery to arise from the internal mammary; two such cases have been reported to date, both in adult patients. Here we present a third case of EPS with arterial supply from the internal mammary successfully treated with video-assisted thoracoscopic resection in a 9 month old infant.

## Background

Extrapulmonary sequestration is a rare congenital lung disease with variable presentation. Treatment is surgical resection, and initial experiences universally employed thoracotomy. Thoracoscopy is being increasingly utilized for pulmonary resections for many etiologies in both pediatric and adult patients, including pulmonary sequestration [1–3]. Here we present a case of extralobar pulmonary sequestration with vascular supply from the left internal mammary artery resected thoracoscopically without surgical complication in a 9 month old child.

## Case presentation

**Fig. 1:**
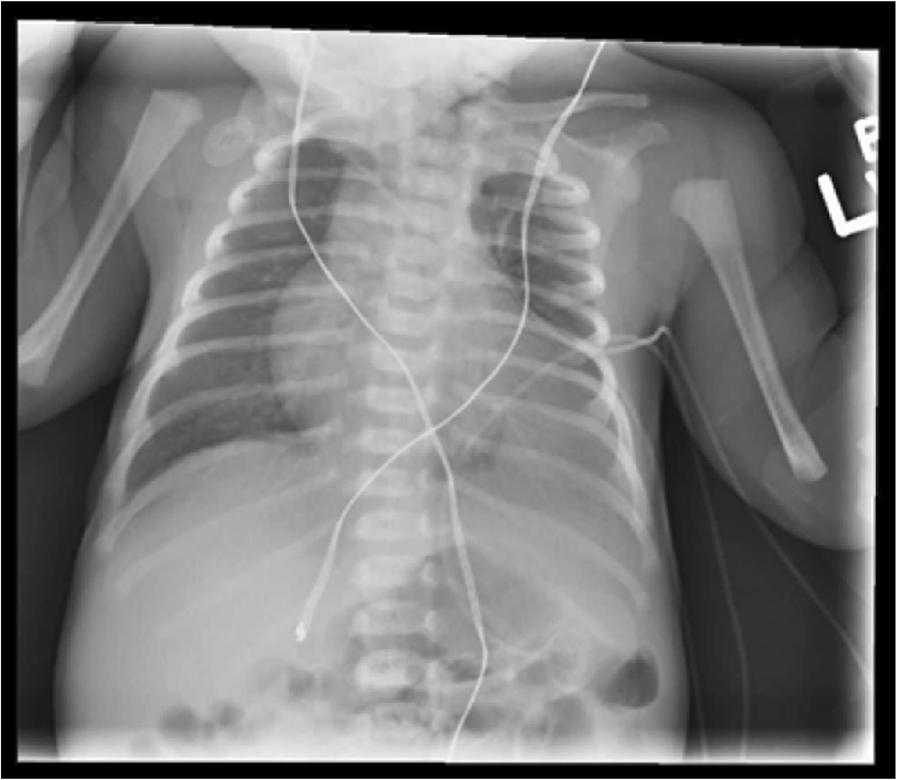
Pre-operative Chest X-Ray. Initial presenting chest X-ray demonstrating left lower lobe opacification

**Fig. 2:**
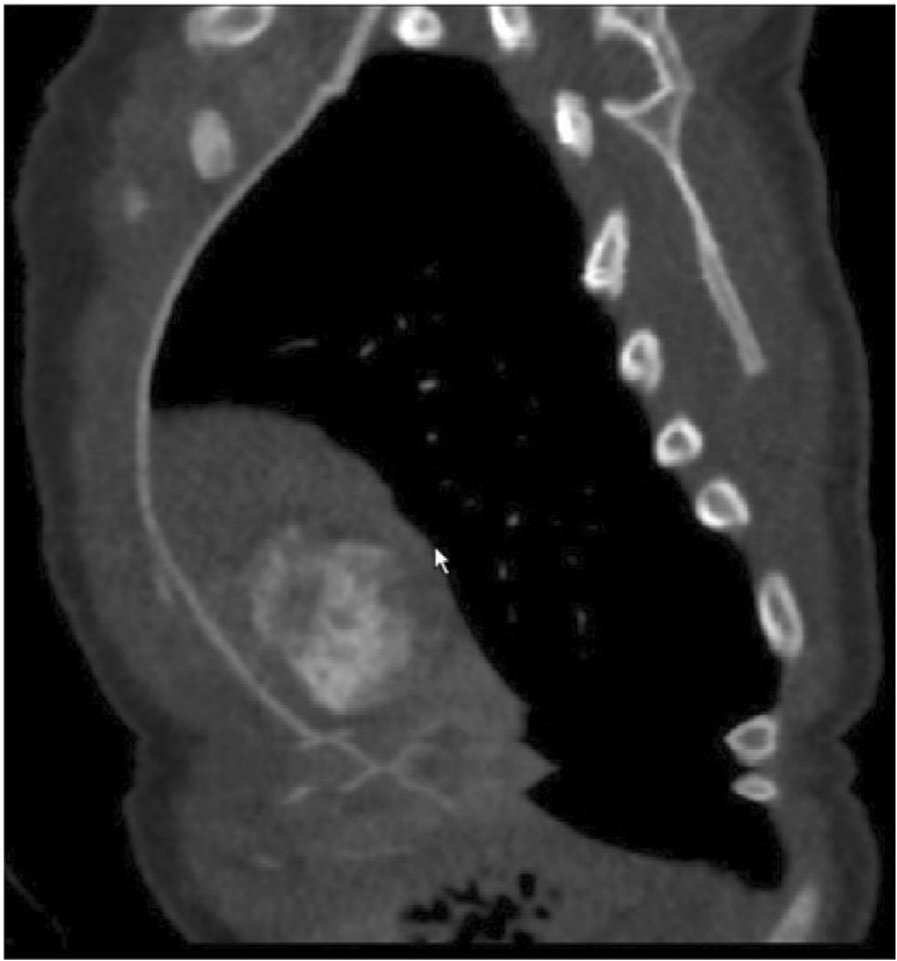
Pre-operative CT angiogram. Sagittal view obtained from pre-operative CT angiogram demonstrating left lower lobe sequestration with blood supply from the left internal thoracic artery

**Fig. 3:**
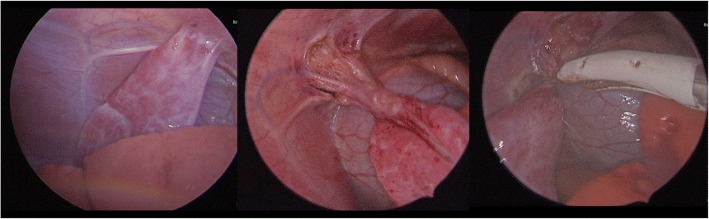
Intra-operative Findings. Steps of left lower lobe sequestration resection off of the chest wall with a view of the left internal thoracic artery

**Fig. 4:**
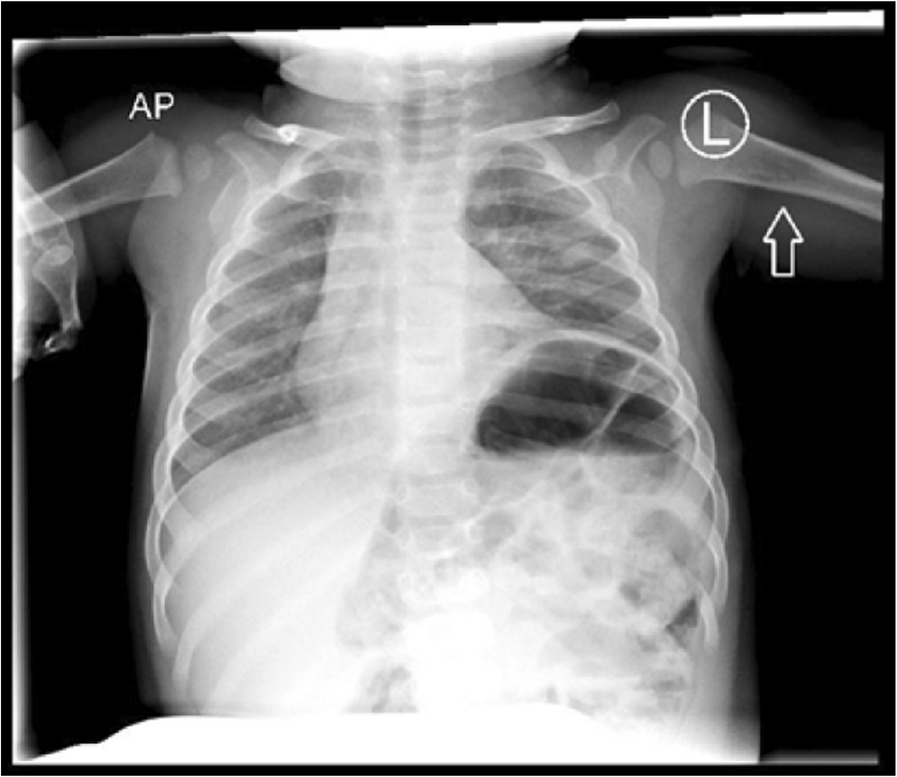
Post-operative Chest X-Ray. Persistent asymptomatic left hemidiaphragm elevation demonstrated on post-operative chest X-ray

Initial presentation of this patient was on day 3 of life; he is a former 37 week, 4 day gestation infant requiring treatment of hypoglycemia secondary to maternal gestational diabetes after emergency C-section delivery due to decreased fetal activity. Routine plain radiograph was obtained for confirmation of umbilical vein catheter placement. This revealed opacification of the left lung fields and was initially viewed as consistent with pneumonia (Fig. 1). A diagnostic thoracic ultrasound was performed which demonstrated the left lobe of the liver corresponding to the area of opacification with concern for left hemidiaphragm eventration versus congenital diaphragmatic hernia. As the patient was hemodynamically normal with no respiratory symptoms, repeat imaging in 1–2 months was recommended. The patient re-presented at 2 months of age with respiratory synctitial virus (RSV) bronchiolitis which resolved with supportive care; interval chest X-ray at this time demonstrated a stable opacity. At 9 months of age an outpatient CT angiogram of the chest was obtained which demonstrated extrapulmonary versus intrapulmonary sequestration with a systemic feeding vessel from the left internal mammary artery (Fig. 2). He underwent elective video assisted thoracoscopic resection of the lesion, which was found to be a left lower lobe extrapulmonary sequestration without communication to the tracheobronchial tree with feeding vessel off the internal mammary as seen on pre-operative CT angiogram. (Fig. 3). Diaphragmatic plication was considered, but not performed due to the asymptomatic nature of this patient’s disease. Intubation was achieved with a single lumen cuffed 4–0 endotracheal tube. Complete resection was achieved without complication utilizing two 5 mm ports, a 30 degree thoracoscope, and an enlarged port site for removal of the specimen. Histology was consistent with extrapulmonary sequestration. He recovered uneventfully and was thriving at his 1 month post-operative visit, notably with persistent but asymptomatic hemidiaphragm elevation on chest X-Ray (Fig. 4).

## Discussion and conclusions

Extrapulmonary sequestration occurs when nonfunctional lung tissue develops from the anterior foregut as an accessory lung bud and remains in the abdomen or chest without connection to the tracheobronchial tree. Its incidence is approximately 0.1% in the general population and in 10–15% of cases the tissue is extralobar (as opposed to intralobar). Those cases that occur in an extralobar fashion tend to associate with other congenital anomalies and on average present earlier, and with increased severity, when compared to intralobar presentations [1, 2].

Definitive treatment of extrapulmonary sequestration is resection. Movement toward a minimally invasive, thoracoscopic approach in the management of surgical lung disease has been widely adopted [3, 4]. In the pediatric population, and particularly in those patients with congenital anomalies that may relatively contraindicate single lung ventilation, the utilization of minimally invasive techniques remains surgeon-dependent. Pediatric patients present the technical challenge of small size, narrow intercostal spaces, and associated difficulty visualizing intrathoracic structures utilizing a minimally invasive thoracoscopic approach [5].

Recent reviews of thoracoscopy versus thoracotomy in a pediatric population found similar outcomes in case matched control studies [6–8]. Pre-operative imaging is integral to the safety and efficacy of these practices. While pulmonary angiography is arguably the gold standard for characterizing extrapulmonary sequestration and other congenital lung disease, CT angiogram in practice has been equivalent and sufficient for exceptional safety and confidence when entering the operating room for a thoracoscopic resection [9]. In this case, the left internal thoracic artery was identified on pre-operative CT angiogram as the systemic feeding vessel. Only two cases with similar anatomy have been reported to date, both in adult patients. The first of these patients presented with the left internal thoracic artery and pulmonary vein as systemic arterial and venous vessels, respectively; the second presented with two feeding arterial vessels, one the right internal thoracic and the other arising from the right renal artery [10, 11]. Here we present a third case with internal thoracic arterial supply, and provide an example of the reliability of pre-operative CT angiography and thoracoscopic techniques for complete resection in an anatomically challenging case in a pediatric patient.

## Abbreviations

CTA: Computed tomographic angiogram
EPS: Extrapulmonary sequestration
